# Flash Burn of the Eyes Caused by High-Voltage Electrical Spark

**DOI:** 10.7759/cureus.12662

**Published:** 2021-01-12

**Authors:** Woo Kyung Lee, Sarah M Barnett, Trilok Stead, Paul R Banerjee, Latha Ganti

**Affiliations:** 1 Emergency Medicine, Coliseum Medical Centers, Macon, USA; 2 Emergency Medicine, Mercer University School of Medicine, Macon, USA; 3 Emergency Medicine, Trinity Preparatory School, Winter Park, USA; 4 Emergency Medicine, Envision Physician Services, Plantation, USA; 5 Emergency Medicine, University of Central Florida College of Medicine, Orlando, USA; 6 Emergency Medicine, HCA Healthcare Graduate Medical Education Consortium Emergency Medicine Residency Program of Greater Orlando, Orlando, USA

**Keywords:** electrical burn, corneal injury

## Abstract

We present a rare case of corneal abrasion with mild eyelid epitheliopathy caused by a high-voltage electrical spark. The case includes emergency department evaluation and subsequent management at the burn center with ophthalmology. The prognosis, in this case, is good, however, the potential severity of high-voltage electrical injuries can be much worse. Prevention strategies for occupational electrical injuries are discussed with an emphasis on proper personal protective equipment (PPE).

## Introduction

Electrical injuries can involve almost all organs of the body, including the central nervous system and the eyes. The severity of the electrical injury depends on the type of current (direct or alternating), the strength of the current (voltage and amperage), duration of exposure, and amount of resistance to flow from body tissues [1-3]. Bodily tissues differ in their resistance to electrical current based on their composition and density. Tissues susceptible to electrical injury from the most to least susceptible are nerve, blood vessel, muscle, skin, tendon, fat, and bone. Damage to these tissues occurs in large part by the conversion of electrical energy into thermal energy [1]. The anatomical inlet and the pathway of the current determine which specific tissues are damaged. The hand is the most common inlet, followed by the head. Burns may be sharply demarcated on the skin when the current penetrates into deeper tissues; however, the absence of external burns does not exclude internal electrical injury [3].

In a retrospective study on electricity induced injuries, 3.1% of patients had ocular manifestations. These incidents most often occurred at voltages greater than 200 V [4]. Ophthalmic manifestations of electrical injury occur in three ways: the direct effect of electrical current passing through the optic nerve and ocular structures, the conversion of electrical energy into thermal energy, leading to thermal damage of ocular tissues, and lastly, tissue ischemia that can be caused by vasoconstriction or cardiac insufficiency [1]. Electrical current reaching both eyes may cause asymmetrical damage due to asymmetric proximity to the route of the current [2].

Numerous ocular manifestations of electrical injury have been reported, but by far the most common is cataract formation. Opacification may occur within hours or days after injury or it can develop months later. In animal studies, the amount of time it took for opacification to occur was found inversely proportional to the severity of the electrical insult. Other well-documented ocular changes include the development of macular holes and optic neuritis [4]. As electrical current initiates its pathway across the orbit, an insult to surrounding tissues often occurs, including conjunctival hyperemia, chemosis, and singe or loss of eyebrows and eyelashes [5]. Severe lid burns can result in trichiasis [6]. When the cornea is involved in an electrical injury, manifestations often include interstitial opacities and loss of corneal epithelial and endothelial layers. In cases of extensive damage, complete opacification, thinning, necrosis, and perforation may occur [4].

While corneal epithelial defects are a rare complication of electrical injury, they are the leading ophthalmic injury seen in the general population [7-8]. The most common cause of a corneal defect is mechanical trauma (e.g. fingernail scratch, the edge of a contact lens, foreign body in the lid, trichiasis or distichiasis, etc.). Chemical exposure, ultraviolet burns, decreased tear production, limbal stem cell deficiency, topical anesthetic abuse, neurotrophic keratopathy, and infection are also well-documented corneal pathologies [7]. Corneal abrasions typically present with acute pain, tearing, blurred vision, photophobia, and a foreign body sensation. Corneal lacerations or foreign bodies may be present. The prognosis is largely dependent on the size of the defect, wound contamination, and the depth of the injury [8].

Emergency treatment of corneal abrasions comprises preventing infection, removing any foreign body, and providing analgesia as the limbal stem cells regenerate the corneal epithelium. Patients should be followed closely by ophthalmology to ensure the resolution of the defect and to monitor for signs of infection [7,9].

## Case presentation

A 30-year-old male electrician presented to the emergency room with electrical and thermal burns to the face and neck occurring about 30 minutes prior to arrival. He attempted to connect two wires at a power box and the wires sent out a large blue arc up to his face with immediate pain to the face, eyes, and neck. He described irritation of the left eye associated with photophobia and blurry vision and a burning sensation under his left armpit, which was raised. Past medical history was significant for anxiety.

**Figure 1:**
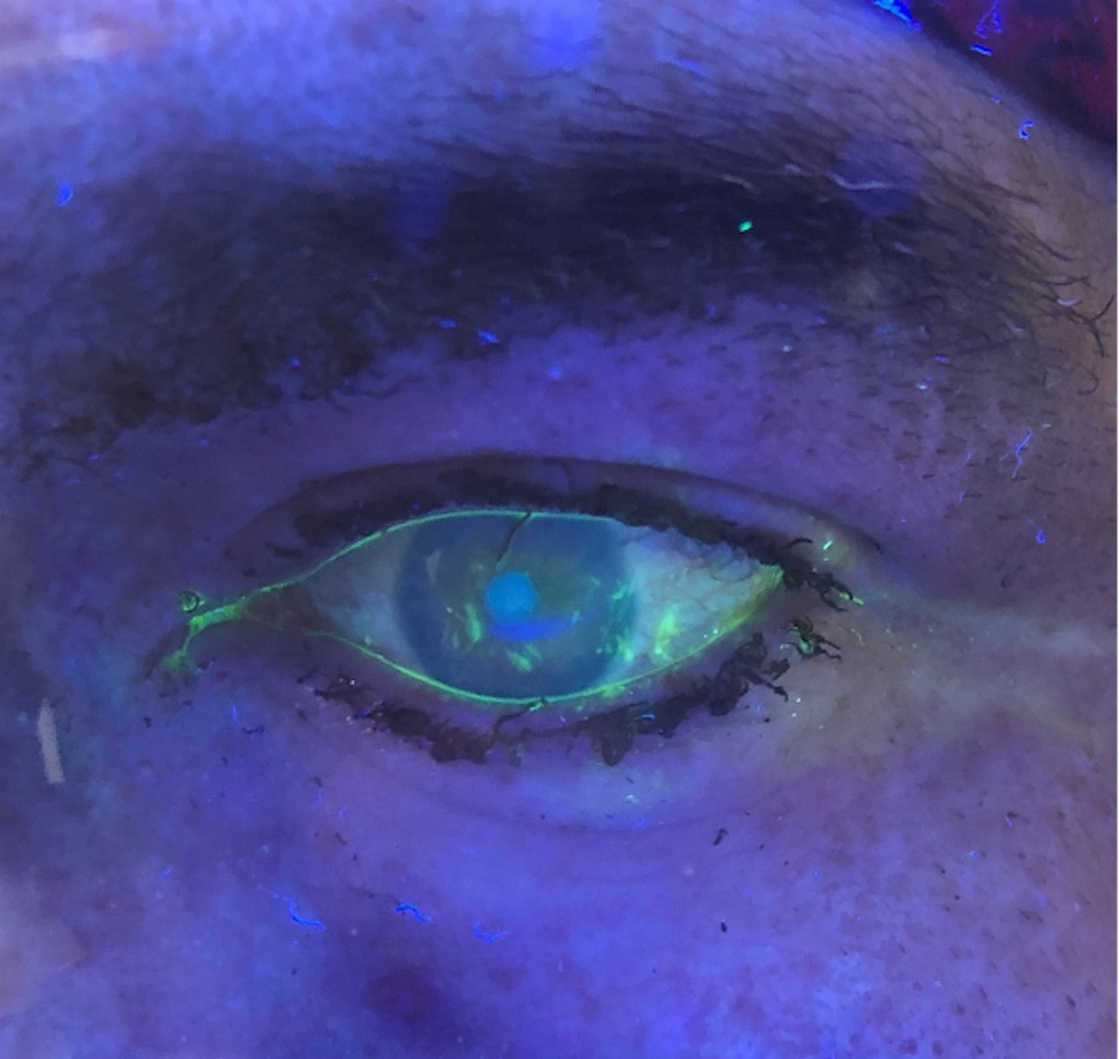
Fluorescein staining of the eye demonstrating corneal abrasion

On examination, heart rate was 66 beats per minute (bpm), O_2_ saturation 97% on room air, respiration 18, and blood pressure 153/78 mmHg. Electrocardiogram revealed normal sinus rhythm at 65 bpm. A focused eye examination revealed singed facial hair and lashes, bilateral conjunctivae and sclerae injection, and eyelid edema. The eyelid opened easily with passive lifting. There was no retained foreign body, and extraocular movements were intact. Wood’s lamp examination with fluorescein staining revealed corneal abrasions (Figure 1).

**Figure 2:**
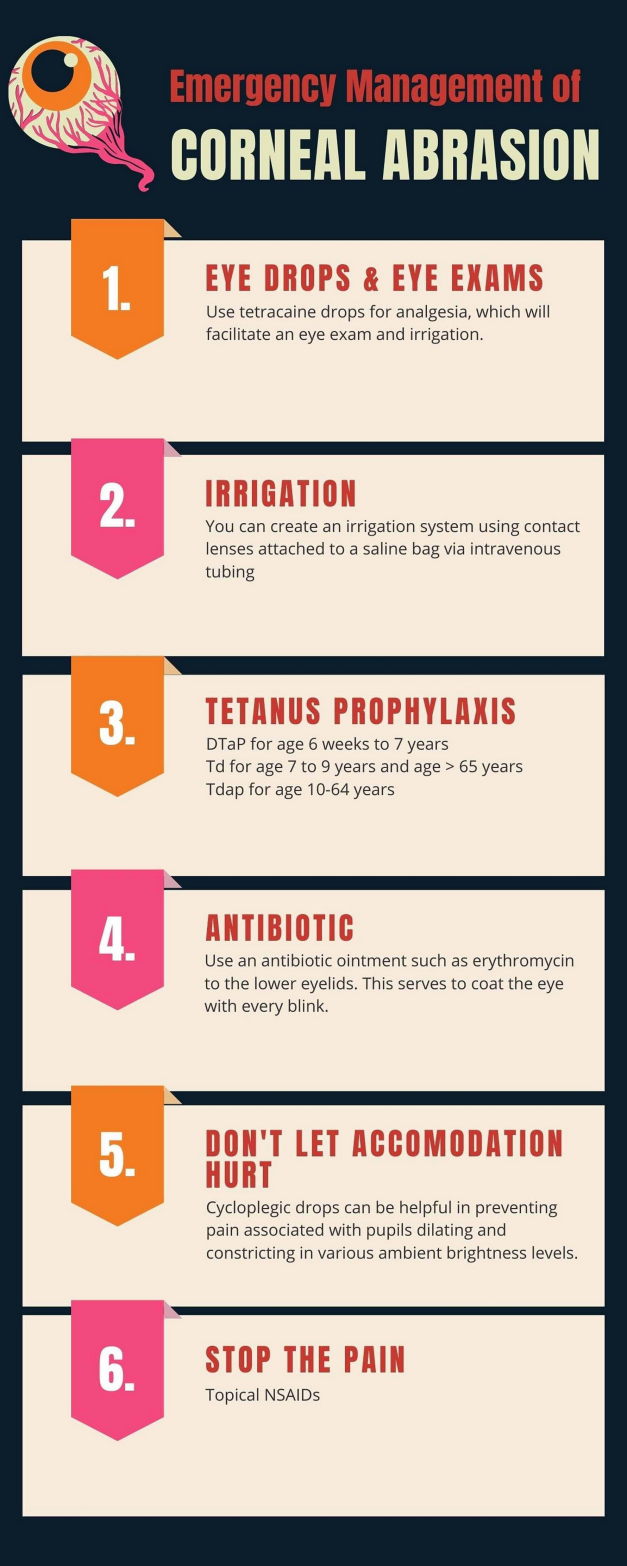
Emergency management of corneal abrasions

There was no evidence of soot in the nostrils. Mucous membranes were moist. The neck was supple with a first-degree burn. A second-degree burn was noted on the left forearm that revealed some hyperemic devitalized tissue that appeared to be separating from the lower dermal layers. Positive peripheral pulses and brisk capillary refill were appreciated. Equal chest rise and fall were noted with inspiration and expiration. The patient’s breathing was non-labored on room air. Based on the history and clinical findings, the injuries were determined to be high-voltage electrical injuries associated with a possible eye injury. Tetanus prophylaxis was administered (Figure 2). The patient was transferred to a burn center for a higher level of care. 

Ophthalmology was consulted at the burn center and the patient was diagnosed with a flash burn of the eyes with left upper and lower eyelid burns and mild surface epitheliopathy. Erythromycin ointment three times a day for 10 days was prescribed. Wounds were seen by a wound nurse, and an antibiotic-impregnated absorbent foam was applied. Burns were categorized as second-degree on the left wrist and first-degree to the face and neck with a total body surface area (TBSA) of 2%. Antibiotic ointment two to three times daily to the face and neck was prescribed and follow up was scheduled at the outpatient burn clinic.

A week later, the patient returned to our emergency department to say thank you for the management. He had a mild foreign body-like irritation sensation on his left eye, but his visual acuity was back to normal. The patient has been following up with the burn center for scar management.

## Discussion

More than 30,000 nonfatal shock incidents occur in the US each year. An additional 300 high-voltage injuries result in fatality. One review of 99 electrical cases that came in over a 10-year period reported that 75.8% of the cases were occupational injuries. There was hand involvement in 60.1% of cases, and head and neck involvement in 58.6% of cases [10].

The range of severity is broad, from multi-system failure to minor cutaneous thermal injury. Corneal abrasions due to electrical injury are rarely reported in the literature, although there is abundant documentation on corneal abrasions in general.

Corneal abrasions from most mechanisms of injury can largely be prevented by wearing protective eyewear. Sunglasses can prevent UV light exposure, and safety glasses can prevent foreign bodies from entering the eye. Electricians, linemen, and wiremen who could be exposed to electric arcs should wear eye protection that is fully dielectric with no metal parts. In some scenarios, a face shield may be needed to protect against electrical arc as well [7,11]. In addition to eye protection, hands and feet should be protected from electrical inlets by wearing rubber insulated gloves and shoes. When working with overhead electrical hazards, such as powerlines, industrial protective helmets should be worn [6].

Doan and co-authors examined 54 workers involved in flash arc incidents to assess levels of protection offered by personal protective equipment (PPE). Approximately half of the workers who suffered electrical burn injuries were not wearing gloves or a face shield with a hard hat. Of the other half, only 18 workers had chosen the correct PPE for the given job. The authors concluded that workers may wear insufficient PPE if they determine there is a low risk of an arc flash event. Arc-rated protective clothing and equipment were seen to provide protection as long as it was selected to match the level of electrical hazard [12].

Along with proper PPE, de-energizing equipment has been shown to provide the best protection against an arc flash [11]. “Lock out, Tag out, Try” is an important safety mantra used by electricians and mechanics before working on equipment. This includes locking out all forms of energy, including mechanical, thermal, potential, and electrical energy; placing an identified tag on each lock; and proceeding to try to turn the equipment on to verify the power is off. The Mine Safety and Health Administration conducted personal interviews with 32 miners who were either arc flash victims or witnesses to an arcing event. Workers who were interviewed overwhelmingly believed that the incidents could have been prevented. Turning off the power source before proceeding to work on the equipment was the most often cited key to prevention [13].

## Conclusions

This case provides an example diagnosis and management of a rare electrical injury. Management in the emergency room or burn center should be conducted with the consultation of ophthalmology to closely follow the extent of corneal epithelial damage and monitor for signs of subsequent evolving ocular damage. Furthermore, this case report highlights the need for raised awareness of electrical hazards and electrical injury prevention. The majority of occupational electrical injuries are preventable by wearing proper PPE and halting the power supply before working on equipment. Better electrical safety training programs and adherence to established electrical safety guidelines can lead to less electrical accidents.
